# Multiplexing Biosensor for the Detection of Extracellular Vesicles as Biomarkers of Tissue Damage and Recovery after Ischemic Stroke

**DOI:** 10.3390/ijms24097937

**Published:** 2023-04-27

**Authors:** Silvia Picciolini, Valentina Mangolini, Francesca Rodà, Angelo Montesano, Francesca Arnaboldi, Piergiuseppe Liuzzi, Andrea Mannini, Marzia Bedoni, Alice Gualerzi

**Affiliations:** 1 IRCCS Fondazione Don Carlo Gnocchi ONLUS, 20148 Milano, Italy; spicciolini@dongnocchi.it (S.P.); vmangolini@dongnocchi.it (V.M.); froda@dongnocchi.it (F.R.); amontesano@dongnocchi.it (A.M.); mbedoni@dongnocchi.it (M.B.); 2 Dipartimento di Medicina Molecolare e Traslazionale, Università degli Studi di Brescia, 25122 Brescia, Italy; 3 Clinical and Experimental Medicine PhD Program, University of Modena and Reggio Emilia, 42100 Modena, Italy; 4 Dipartimento di Scienze Biomediche per la Salute, Università degli Studi di Milano, 20133 Milano, Italy; francesca.arnaboldi1@unimi.it; 5 IRCCS Fondazione Don Carlo Gnocchi ONLUS, 50143 Firenze, Italy; pliuzzi@dongnocchi.it (P.L.); amannini@dongnocchi.it (A.M.); 6 Scuola Superiore Sant’Anna, Istituto di BioRobotica, 56025 Pontedera, Italy

**Keywords:** extracellular vesicles, Surface Plasmon Resonance imaging, biosensor, stroke, biomarkers

## Abstract

The inflammatory, reparative and regenerative mechanisms activated in ischemic stroke patients immediately after the event cooperate in the response to injury, in the restoration of functions and in brain remodeling even weeks after the event and can be sustained by the rehabilitation treatment. Nonetheless, patients’ response to treatments is difficult to predict because of the lack of specific measurable markers of recovery, which could be complementary to clinical scales in the evaluation of patients. Considering that Extracellular Vesicles (EVs) are carriers of multiple molecules involved in the response to stroke injury, in the present study, we have identified a panel of EV-associated molecules that (i) confirm the crucial involvement of EVs in the processes that follow ischemic stroke, (ii) could possibly profile ischemic stroke patients at the beginning of the rehabilitation program, (iii) could be used in predicting patients’ response to treatment. By means of a multiplexing Surface Plasmon Resonance imaging biosensor, subacute ischemic stroke patients were proven to have increased expression of vascular endothelial growth factor receptor 2 (VEGFR2) and translocator protein (TSPO) on the surface of small EVs in blood. Besides, microglia EVs and endothelial EVs were shown to be significantly involved in the intercellular communications that occur more than 10 days after ischemic stroke, thus being potential tools for the profiling of patients in the subacute phase after ischemic stroke and in the prediction of their recovery.

## 1. Introduction

Stroke occurs when a blockage or bleed of the blood vessels either interrupts or reduces the nutrient and oxygen supply to the brain, causing cell death in the brain tissue. The local hypoxia and the consequent neuronal cell death determine signs and symptoms that are proportional to the extent and duration of the blood interruption. Globally stroke has been identified as the second cause of death by the World Health Organization’s Global Health Estimates and the fourth cause of disability for over 65-year-old people [1]. Independently from its cause (either blockage or bleed of a vessel), stroke determines severe disability in about one-third of survivors, with rehabilitation treatment being crucial not only for motor recovery but also for cognitive, respiratory and speech functions, among others.

Although the acute treatment of patients after stroke has highly improved life expectancy, disability remains a high-priority issue and a significant clinical challenge for post-stroke patients. In the acute phase, most drugs and treatments are aimed at normalizing the electrochemical balance of the brain in order to minimize the likelihood of a secondary injury, but once the vital parameters of stroke patients are stabilized, the rehabilitation phase is fundamental to sustaining the spontaneous brain and vessel remodeling. Rehabilitation can be based on physical, occupational, speech, or recreational therapies [1], but must be tailored to the patients’ clinical features in order to achieve the best results. For this reason, the preliminary evaluation at the admission at the rehabilitation hospital should be as exhaustive as possible. Protocols have been validated by multiple national societies, including the Italian Society of Physical and Rehabilitation Medicine (SIMFER) [2], to identify the clinical variables that are considered essential for the establishment of a personalized rehabilitation program including neurological examination and validated clinical scales [3]. Still, a quantitative variable is needed to provide a decision support tool to clinicians, i.e., biomarkers to objectively profile patients, not only in the acute phase but also in the sub-acute phase of the disease in order to identify the optimal treatment. Up to now, most biomarkers evaluated in subacute stroke patients were soluble circulating molecules, such as neurotrophic factors [4], cytokines and others [5], but none of them have currently demonstrated suitability for the objective profiling of patients and for the prediction of patients’ response to treatment.

Extracellular vesicles (EVs) are known to be carriers of bioactive molecules involved in the pathophysiology of stroke, in particular in the inflammatory, regenerative, and reparative mechanisms activated after stroke injury. It was previously reported that specific EV subtypes, such as platelet-derived and T-cell-derived EVs, are associated with stroke severity and short- and long-term outcomes [6]. Great attention has been also devoted to miRNAs, which were correlated with the susceptibility to ischemic stroke [7] and they were proven to be involved in the EV-mediated intercellular communication that follows a stroke event, suggesting a potential as diagnostic/ prognostic biomarkers as well as therapeutic tools [3,8,9]. However, little attention has been dedicated so far to the EVs released by the brain and blood vessels during the plasticity and remodeling after stroke [3]. EVs may represent potential valuable biomarkers for stroke as those vesicles that circulate in blood can mirror the processes occurring in the brain as well as in all of the other body organs directly or indirectly involved in the reparative mechanisms activated after stroke.

In the last years, a plethora of high throughput techniques has been reported for the single and bulk characterization of EVs, with the aim of overcoming the current obstacles in EV detection and accelerating the translation of EV research to clinics [10,11,12,13,14,15,16,17].

Some of the most promising are nano/high flow cytometry and fluorescent nanoparticles tracking analysis (f-NTA) for the determination of EV size and concentration, Single-Molecular array (SiMoA) and Single Particle Interferometric Reflectance Imaging Sensor (SP-IRIS) whose detection model is based on fluorescence. Raman, surface-enhanced Raman spectroscopy or infrared analysis are other techniques that are used for the determination of EV composition in bulk without specific markers [11]. In this study, we have taken advantage of a multiplexing Surface Plasmon Resonance imaging (SPRi) based biosensor [18] that allows the detection of low concentrations of multiple biomarkers concomitantly in a label-free manner analyzing intact EVs and the study of the expression of molecules on EV surface due to the intrinsic SPR enhancing property of the EV membrane. The SPRi method combines multiplexing analysis with the imaging capability that enables the visualization of the working area in real time, allowing the EV phenotyping with a limited amount of samples and reagents. Moreover, the potential presence of contaminants in the EV samples that we obtained by size exclusion chromatography (SEC), does not represent a limitation in our protocol because, during the SPRi experiment, the sample flowing on the surface of the biochip undergoes an additional purification step detecting EVs specifically according to their interactions with ligands. These features make SPRi adaptable for the development of SPRi-based diagnostic and prognostic tools. In particular, we have now used the SPRi biosensor to investigate the role of EVs in the evolution of the clinical status of post-stroke patients, with a particular focus on blood small EVs (<200 nm) [19] carrying markers of brain and vessel origin. The biosensor was adapted and optimized for the evaluation of the extent of damage and for the investigation of the reparative mechanisms activated in stroke patients that might be involved in the differential response to the rehabilitation treatment and in the consequent recovery. After the isolation of EVs from serum samples, the correlation between EV-associated markers and clinical scales was verified in order to profile patients and assess the predictive potential of EVs. In parallel, inflammatory cytokines and the brain derived neurotrophic factor (BDNF) were measured in serum in accordance with the reported literature [5,20].

To the best of our knowledge, this is the first time that such a wide panel of biomarkers (10 brain and non-brain-associated proteins) has been investigated in the blood EVs from stroke patients in the sub-acute phase after the event. The reported results confirm the considerable applicability and versatility of the proposed SPRi biosensor and, specifically for stroke, the tremendous clinical potential of EVs as predictive biomarkers of recovery after injury when the rehabilitation strategy needs to be defined, even days after the lesion. Finally, the present study is a pioneering report in rehabilitation medicine, proposing EVs as rehabilitation biomarkers, i.e., biomarkers of patients’ response to rehabilitation, paving the way for further studies looking for precision medicine applications in the rehabilitation field.

## 2. Results

### 2.1. Stroke Patients Profiling

**Figure 1:**
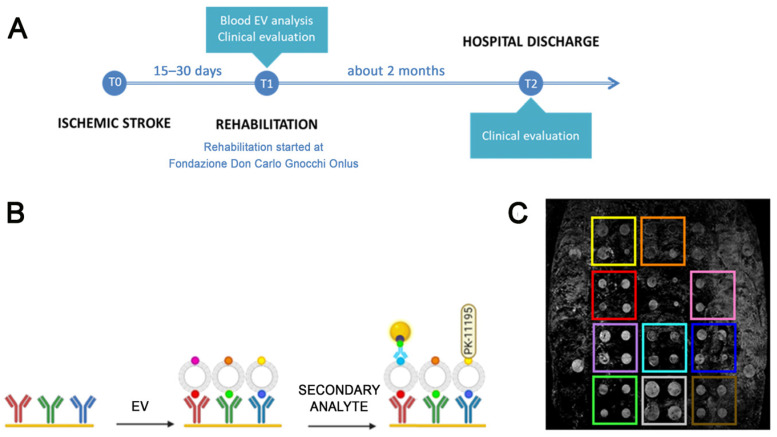
Experimental design. (**A**): Schematic representation of the experimental setting of the present study. (**B**). Schematic of the analysis performed by means of the SPRi biosensor: first the gold biochip is functionalized with antibodies or ligands specific for EVs from selected cell source; then, specific EV subfamilies are immobilized on the biochip thanks to tissue related markers; finally, the immobilized EVs are characterized with a secondary injection of antibodies conjugated with gold nanoparticles to enhance the SPRi signal, or synthetic ligands specific for surface molecules, in this case the TSPO ligand PK-11195. (**C**): Representative CCD differential image of the SPRi chip surface where the 12 families of ligands spotted on the biochip can be visually detected.

In the present study, a cohort of 19 ischemic stroke patients was successfully recruited at IRCCS Fondazione Don Carlo Gnocchi (Milan, Italy). All subjects were clinically profiled using the Modified Barthel Index (MBI) [21], an established clinical scale for the evaluation of a patient’s disability, at admission and discharge, i.e., after completing the assigned rehabilitation protocol (about 2 months after admission) (Figure 1A). Twenty age and sex matched volunteers (CTRL) in good health status were enrolled as the control group, too. Table 1 summarizes the characteristics of stroke patients and healthy controls included in the study.

**Figure 2:**
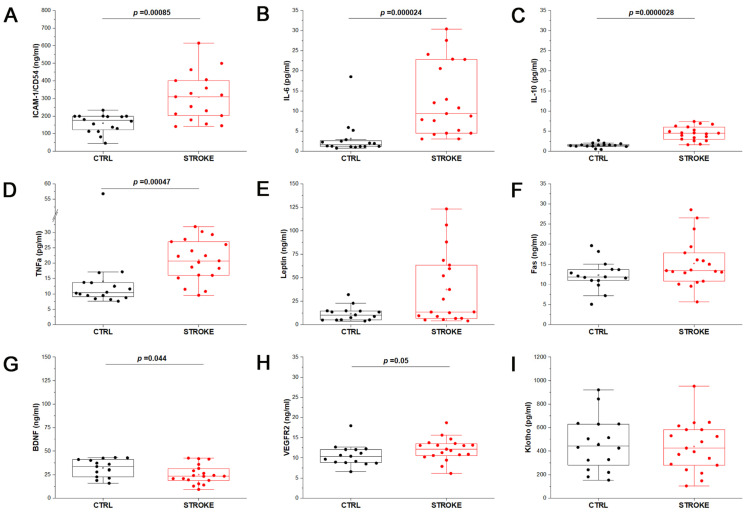
Stroke patients profiling. Box plots represent the quantification of soluble mediators of inflammation and regeneration by enzyme-linked immunosorbent assay on serum samples of control samples (CTRL; *n* = 16) and stroke patients (STROKE, *n* = 19), by means of ELISA plates or ELLA automated system. The results obtained for ICAM-1 (**A**), IL-6 (**B**), IL-10 (**C**), TNFα (**D**), Leptin (**E**), Fas (**F**), BDNF (**G**), VEGFR2 (**H**), Klotho (**I**) are reported. Mann–Whitney test was applied to all measurements to compare the distribution of the two experimental groups. The *p*-value is reported when the two distributions resulted statistically significant (*p* < 0.05).

To better profile the recruited stroke patients, as well as volunteers of the control group, we performed the quantification of the serum levels of the main cytokines (IL-6, IL-10, TNFα) and soluble factors (ICAM-1, Leptin, Fas, BDNF, VEGFR2) known to be involved in the pathogenesis and in the brain plasticity that occurs after stroke [5]. Comparing CTRL and ischemic stroke samples, higher levels of the soluble Intercellular Adhesion Molecule 1 (ICAM-1, also known as CD54; Figure 2A), as well as IL-6 (Figure 2B), IL-10 (Figure 2C), TNFα (Figure 2D) and Leptin (Figure 2E) were observed, even though only ICAM-1, IL-6, IL-10 and TNFα proved to be significantly increased in the serum of stroke patients compared to CTRL (*p* < 0.001, Mann–Whitney test). Thanks to the ELLA instrument, also Fas (Figure 2F), BDNF (Figure 2G) and VEGFR2 (Figure 2H) were quantified in stroke and CTRL samples, observing a slight increase in VEGFR2 concentration in stroke compared to CTRL (*p* = 0.05, Mann–Whitney test), while BDNF was significantly decreased in stroke patients (*p* = 0.044, Mann–Whitney test). Finally, the anti-age protein Klotho, associated with vascular regeneration, was also quantified by ELISA assay. Klotho is known to be a bioactive molecule transported in blood both in soluble and EV-associated form [22] and to play a key role in the cross-communication between muscles and brain; it was proposed as a predictor of functional outcome in acute stroke patients [23]. In the considered samples, no significant difference in the concentration of serum Klotho was observed between the two experimental groups (Figure 2I). Data from ELISA and ELLA assays are reported in Supplementary Table S1.

### 2.2. EV Physico-Chemical Characterization

**Figure 3:**
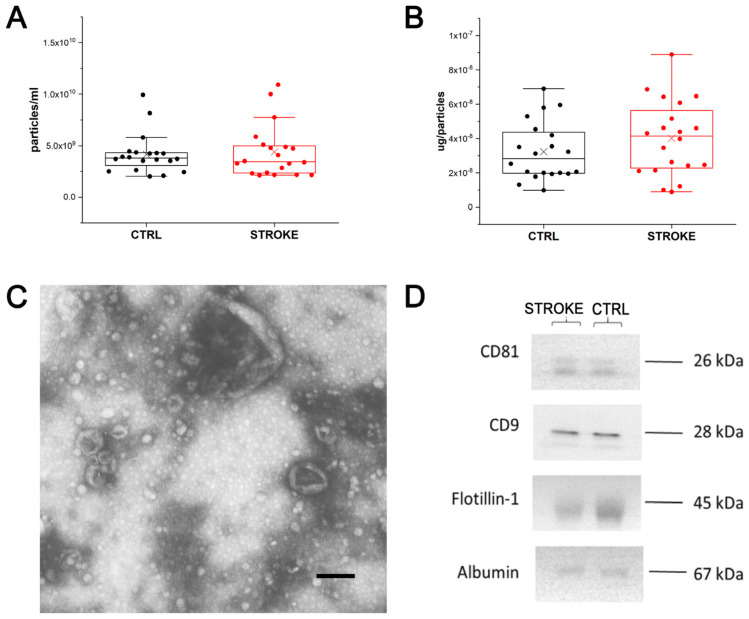
Extracellular Vesicle characterization. (**A**): Box plot reporting the EV concentration obtained by NTA analysis on EV preparations from healthy controls (CTRL; black) and stroke patients (STROKE; red). Each dot represents the mean value obtained for each subject after five acquisitions, whereas X represents the mean value for each group. (**B**): Box plot reporting the μg of proteins per particles obtained for each EV preparations from healthy controls (CTRL; black) and stroke patients (STROKE; red). The value was obtained dividing the μg of proteins measured by BCA assay for the concentration of particles obtained by NTA. Each dot represents the mean value obtained for each subject, whereas X represents the mean value for each group. (**C**): Representative TEM image obtained from the analysis of EV preparations from healthy controls and stroke patients. Black bar: 200 nm. (**D**) Western Blot analysis performed on EVs from both experimental groups revealing the presence of Flotillin-1, CD9 and CD81 as well as the faint signal from Albumin, co-isolated with EVs from serum.

Small EVs were isolated from all considered serum samples by the SEC isolation method. The NTA demonstrated that the EV concentration was similar in samples from stroke patients and CTRL (Figure 3A), as well as their dimensions (mean size: CTRL: 150 nm ± 24.5; STROKE 147.9 nm ± 25.7). Considering the amount of protein per particle, it was found to be increased in stroke patients compared to CTRL (Figure 3B), even though it is not statistically significant (Mann–Whitney test). Data are reported in Supplementary Table S2.

In Figure 3C, EVs with typical cup-shaped morphology and dimensions are shown in a representative Transmission Electron Microscopy (TEM) image obtained from the considered samples. Western blot was also performed to detect some of the main protein markers of EVs as suggested by MISEV [19]. Flotillin-1, CD9 and CD81 were chosen as EV markers, whereas albumin was selected as a non-EV marker. As shown in Figure 3D, the main markers of EVs were detected in both stroke and CTRL samples with comparable intensities. Additionally, albumin was, as expected, found to be co-isolated with small serum EVs.

### 2.3. SPRi Analysis

**Figure 4:**
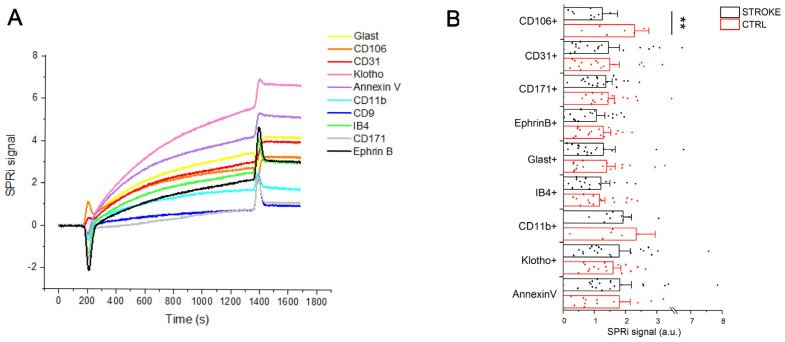
Extracellular Vesicle detection by SPRi biosensor. (**A**): SPRi sensorgram related to the injection of 500 μL of EVs (40 μg/mL) on a chip with an array of 12 families of ligands (four spots of each family). Each curve is the average of the signal collected on the four spots of the same ligand. Each signal is the subtraction between the signal obtained on the specific ligand family and the signal obtained on the negative ctrl family (anti-IgG) spotted on the same chip. (**B**): SPRi intensities (average + standard error) related to the injection of EVs of 19 ischemic stroke patients and 20 CTRL subjects. The signals are normalized for the intensities collected on the anti-CD9 family spotted on each SPRi chip following a previously reported protocol [24]. ** = *p* < 0.05 after Mann–Whitney test.

To characterize the populations of small EVs that circulate in the blood of stroke patients, an SPRi-based biosensor was used. The considered SPRi biosensor was first applied to the evaluation of blood EVs in 2018 [18] and further demonstrated to be suitable for the characterization of human and murine EVs from brain and non-brain cells [22,24,25]. In the present study, the biosensor has been further implemented to recognize up to 12 ligands (including one positive and one negative control) and 10 EV families simultaneously, with a single injection of pre-isolated EVs (Figure 1C). Specific ligands for vesicles derived from endothelial cells (CD31+, CD106+), neurons (CD171+, Ephrin B+), microglia (IB4+, CD11b+) and astrocytes (Glast+) were spotted on the surface of the functionalized gold biochip as well as a marker for aging (Klotho+) and apoptosis (Annexin V binding to phosphatidylserine enriched EVs). The detection of all subfamilies (both brain and non-brain derived EVs) was successful in all considered samples. The relative amount of EVs circulating in the serum of ischemic stroke patients and control subjects showed intragroup variability that was not correlated with either age or sex of recruited subjects. The typical SPRi sensorgram obtained after the injection of isolated small EVs on the biosensor is shown in Figure 4A.

The graph shows the multiplexing ability of the SPRi sensor to detect the binding of multiple EV families of interest taking advantage of specific markers related to their parental cell and following it over time. Indeed, each signal is related to the interaction between the ligand spotted on the chip and the EVs injected over the chip. For each sample, the same amount of proteins calculated by the BCA assay was injected.

The SPRi signal intensities collected at the end of the injection of each EV sample are indicative of the relative amount of each EV population. Therefore, we compared the SPRi signal intensities related to the injection of EV samples of stroke patients with the ones related to CTRL subjects. In Figure 4B, the value of reported SPRi signals was normalized for the total CD9+ EVs (i.e., the SPRi signals collected on anti-CD9 spots, where CD9 was used as a general marker of small EVs) for their relative quantification.

Comparing the results obtained in the two experimental groups, we observed variations in the level of circulating EVs released by both brain and non-brain cells. In particular, samples from ischemic stroke patients showed an increase in circulating CD106+ (+83.4%), CD31+ (+3.1%), CD171+ (+5.1%), Ephrin B+ (+21.5%) and CD11b+ (+21.8%) EVs, whereas we observed a decrease in the circulating IB4+ (−6.6%) and Klotho+ (−10.7%) compared to control subjects. Due to the intragroup variability of the results, only the increase in CD106+ EVs was found statistically significant (*p* = 0.036, Mann–Whitney test). No variations were observed in the relative amount of Annexin V binding EVs. Data are reported in Supplementary Table S3.

**Figure 5:**
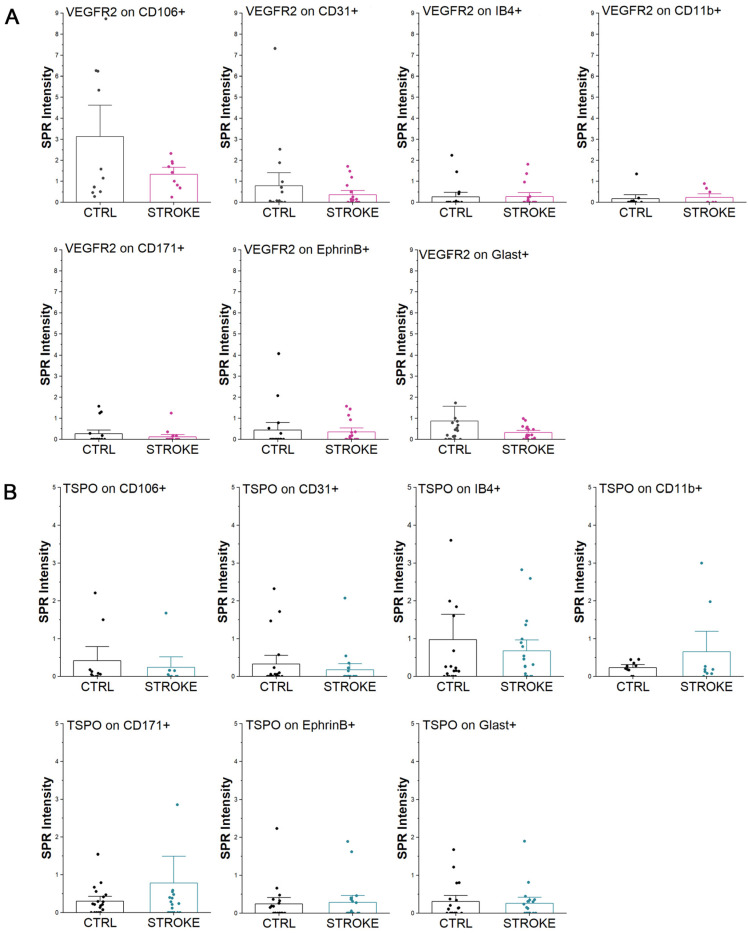
Extracellular Vesicles characterization by SPRi biosensor. SPRi signals obtained on the EVs immobilized on the biosensor by secondary labeling with VEGFR2 antibody conjugated with GNPs (**A**) or with the TSPO ligand PK-11195 (**B**). The signals obtained on EVs from healthy controls (CTRL) and ischemic stroke patients (STROKE) are reported.

To investigate more specifically the presence and expression of markers of regeneration and plasticity associated with EVs, we performed a secondary characterization of the immobilized EVs, by injecting a specific antibody conjugated to gold nanoparticles (GNPs) after EV immobilization on the chip (Figure 1B). Starting from the observed variation in the VEGFR2 levels in serum (Figure 2H), a specific anti-VEGFR2 antibody conjugated to GNPs was used to verify if the variation could be related to the expression of the receptor on the specific subfamilies of EVs bound on the biosensor. The analysis revealed that in stroke patients VEGFR2 was decreased in most of the considered vesicles (CD106+, CD31+, CD171+, Ephrin B+, Glast+), but it was increased in microglia-derived vesicles (IB4+ and CD11b+) (Figure 5A). Nonetheless, for all of the considered markers, no statistically significant variation was found compared to the control samples, demonstrating that the increase in VEGFR2 quantified by immunosorbent assay (Figure 2H) was not referring to the EV-associated receptor.

On the same immobilized vesicles, we explored also the expression of Translocator Protein (TSPO) receptor which is known to be a marker of neuroinflammation, playing an important role in glial activation and neuronal cell death in many central nervous system diseases and injuries [26]. The results shown in Figure 5B demonstrate the controversial expression of TSPO receptor on the surface of circulating EVs. Indeed, in neuron-derived EVs of stroke patients, TSPO was found to increase in CD171+ EVs, but not in the Ephrin B+ vesicles. Similarly, focusing on microglia-derived EVs, TSPO was found more abundant on CD11b+ EVs in stroke patients than CTRL, but not on IB4+ EVs. Considering EVs released by endothelial cells in stroke patients, TSPO was diminished both on CD106+ and CD31+ EVs, without statistical significance compared to controls (Mann–Whitney test). No differences were observed on Glast+ EVs. Data are reported in Supplementary Table S4.

### 2.4. Correlation Study

To evaluate the ability of the proposed EV-associated biomarkers to profile stroke patients, describe their lesions and predict their recovery after rehabilitation, a correlation study was performed. First of all, it was verified that the considered variables were not related to differences in age or sex in both stroke patients and control subjects. A correlation was observed between the amount of CD11b+ EVs and the time from the last stroke event, suggesting the dynamic response over time of microglia that follows the ischemic stroke lesion. Then, we tested whether the SPRi-data were correlated to the patients’ profile defined by the MBI score that was calculated both at the admission (measure of functional independence) and at discharge (measure of recovery) from Fondazione Don Gnocchi.

In the considered cohort of ischemic stroke patients, the univariate Spearman test showed that there is a negative correlation between the expression of VEGFR2 on CD11b+ EVs (*p* = 0.044) and the MBI at admission (i.e., higher levels of VEGFR2 with lower MBI score, thus increased stroke severity and brain damage). On the contrary, there was a positive correlation between MBI at admission and the levels of TSPO receptor on both endothelial EVs (CD106+, *p* = 0.048; CD31+, *p* = 0.035) and in general on small CD9+ EVs (*p* = 0.049), suggesting that the overexpression of TSPO on EVs might indicate a better health status in ischemic stroke patients. In line with these results, the activation of endothelial cells and their overexpression of TSPO receptor was proven to be a predictor of better recovery after rehabilitation, with the amount of TSPO on CD31+ EVs being positively correlated with MBI at discharge, around 2 months after the stroke event (*p* = 0.001, Univariate Spearman test). Considering microglia, SPRi analysis of EV biomarkers brought to light that the relative amount of CD11+ EVs in the serum of ischemic stroke patients correlated with a better MBI at discharge, i.e., with good recovery (*p* = 0.004, Univariate Spearman test). Besides, the crucial involvement of microglia-derived EVs in the recovery of stroke patients is supported also by the positive correlation between the increased expression of VEGFR2 on IB4+ EVs and MBI at discharge (*p* = 0.015, Univariate Spearman test). A summary of the results of the correlation study are reported in Table 2.

**Figure 6:**
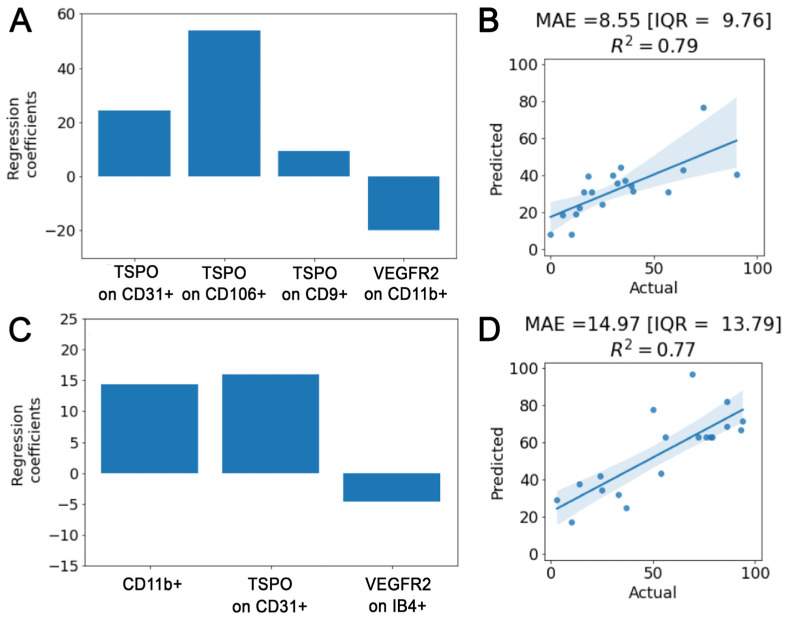
Correlation study. β regression coefficients of the Elastic-Net multivariate analysis for, respectively, the admission (**A**) and discharge (**C**) MBI models. Actual versus predicted plot with median absolute value [IQR] and R^2^ coefficient for, respectively, the admission (**B**) and the discharge (**D**) models.

These results were confirmed by means of 5-fold cross-validated Elastic-Net multivariate regressions. In particular, when considering admission MBI levels as the outcome: (i) the expression of VEGFR2 on CD11b+ EVs, (ii) the levels of TSPO receptor on both endothelial EVs (CD106+ and CD31+) and (iii) on small CD9+ EVs entered the multivariate analysis. None of these descriptors, except for TSPO levels on CD106+, retained their significance after the multivariate analysis, but regression coefficients (averaged across the 5-folds) maintained the trends of the univariate analysis. In particular, higher levels of TSPO receptor on endothelial (CD106+ and CD31+) and small (CD9+) EVs and lower levels of VEGFR2 expression on CD11b+ EVs pointed toward a better health status (Figure 6A).

On the other hand, (i) the total amount of TSPO receptor on CD31+ EVs, (ii) the expression of VEGFR2 on IB4+ EVs and (iii) the relative amount of CD11+ EVs in the serum entered Elastic-Net when targeting discharge MBI as the outcome. Within this analysis, confirmation of the significant positive independent correlation with the discharge MBI of the total amount of TSPO on CD31+ and of the relative among of CD11+ EVs was found in all cross-validation folds (Figure 6C).

The cross-validation accuracy obtained is 8.55 [IQR = 9.76] and 14.97 [IQR = 13.79] points for the admission and discharge MBI estimation with an R^2^ of 0.79 and 0.77, respectively (Figure 6B,D).

## 3. Discussion

In the present study, we have characterized the small EVs that circulate in the blood of ischemic stroke patients in the sub-acute phase of the disease, i.e., more than 10 days after the event. This is indeed a challenge and urgent clinical need in rehabilitation medicine, as rehabilitation treatment needs to be finely tailored to the personal need of stroke patients once the acute and life-saving treatment has been completed. Taking advantage of a multiplexed SPRi-based biosensor we were able to effectively detect multiple subpopulations of EVs by means of specific tissue markers expressed on the vesicle surface. The relative amount of different brain (expressing markers typical of neurons, astrocytes, and microglia) and non-brain (expressing markers typical of endothelial cells) families of small EVs were evaluated in the blood of ischemic stroke patients at admission to the rehabilitation program. In parallel, patients were biochemically profiled by the quantitation of inflammatory mediators (such as cytokines and leptins), BDNF and Klotho, which were already reported in the literature as potential predictors of stroke recovery [5,23], as well as with the most widely used clinical scale for the evaluation of disability in stroke patients, i.e., the modified Barthel index (MBI). The evaluation of the disability by means of MBI was also performed at patients’ discharge, and at the end of the rehabilitation program, and it was used as a clinical measure of recovery after stroke.

In recent years, our knowledge about the involvement of EVs in the events occurring after ischemic brain injury has exponentially increased, as well as the potentialities of EVs such as therapeutics and amplifiers of brain repair and improvement of recovery after stroke [27]. Despite the great efforts made to find stroke biomarkers to be used to improve the patient’s classification and treatment, currently, no sensitive molecule has been identified. The reasons might relate to the heterogeneity of stroke and the complexity of the ischemic cascade that cannot be easily summarized by a single biomarker. Indeed, we believe that a panel of biomarkers might better reflect the pathophysiology of stroke [28]. The herein-reported data support the emerging involvement of EVs in the subacute processes of response to injury and neuroplasticity that follow the stroke lesion [3] and demonstrate the crucial role of endothelial-derived EVs and microglia-derived EVs in the events that follow the ischemic stroke injury, bringing new insights in the mechanisms that impact the outcome of rehabilitation and treatment.

In line with previously reported literature [5,29], we observed that inflammation is a key factor in the pathogenesis of stroke. In parallel to the profiling of patients by means of cytokine quantitation, as already reported in the literature for acute stroke, we evaluated microglia-derived EVs that significantly modify their presence in the blood of ischemic stroke patients in relation to the time spent between blood withdrawal and stroke event, even in the sub-acute phase of the disease. Indeed, EVs from activated microglia (CD11b+) were increased in the serum of the considered subjects, compared to controls, and they were shown to overexpress VEGFR2 in case of severe stroke damage. Moreover, in the sub-acute phase, the sustained response of microglia to the stroke injury and the consequent high blood levels of CD11b+ EVs seem to be predictive of a better recovery after stroke, measured at discharge by MBI. On the contrary, based on previous literature, cytokines are known to reach a steady state in the subacute phase after stroke, being informative mainly in the acute phase, i.e., immediately after the event [30]. However, in the considered cohort, ICAM-1, IL-10, IL-6 and TNF-α serum levels showed a significant trend in the increase. These results are in accordance with literature findings on the increase in TNF-α serum levels of ischemic stroke patients as a part of the acute phase response that occurs during stroke pathogenesis [31,32]. Indeed, the neuroinflammatory response is a dynamic process and the levels of the soluble mediators are known to be fluctuant and strictly related to the observational time point [29], especially in the acute phase of the disease. As an example, it was previously reported that baseline levels of soluble FasL are lower immediately after ischemia compared to healthy controls, but they return to normal levels after 24 h [33]. This is in agreement with previous data from mass spectrometry analysis of circulating EVs of acute ischemic stroke patients where an overall elevation in the inflammatory proteins, especially the acute-phase proteins, was observed in circulating EVs from patients with acute stroke [34], even though we did not register any significant increase in the concentration of circulating vesicles in the sub-acute phase.

Similarly, BDNF was reported to be a valid neuroimmune mediator of stroke that could predict the prognosis of the patients. Low levels are related to poor prognosis [5,20] whereas increased BDNF can be induced by physical exercise, thus enhancing the prognosis even in chronic post-stroke subjects [5,35]. In our cohort, BDNF was only slightly altered in subacute stroke patients compared to controls. This might be due to the time passed between the event and the analysis or to the specific clinical features of the selected experimental group, still in our setting BDNF does not seem to be informative about the patients’ response to injury, nor predictive of their recovery having no correlation with the MBI value evaluated both at admission and discharge.

Similarly, the protein Klotho, both in its soluble and EV-associated form, was not proved to be altered in stroke patients compared to CTRL. Klotho is a pleiotropic protein that was proven to play a protective role against ischemic brain injury [36] and to circulate in high concentrations in the plasma of acute stroke patients, correlating also with better functional outcomes [23]. Unexpectedly, in the considered subjects, despite the physical exercise performed during the rehabilitation protocol that was expected to stimulate the release of Klotho in blood [37], we did not detect any variation in the serum concentration (soluble and EV bound form) and not even in the EV-associated levels.

Looking at the multitude of EVs that circulate in blood after stroke, it was expected to find variations in the populations coming from brain and blood vessels due to the dramatic injury encountered by nervous and vascular tissues during ischemic stroke that leads to disruption of the blood-brain barrier with consequent easy access of vesicles released in the brain to the systemic circulation. In the reported experimental setting, we chose to examine the two main resident cell types involved in the brain tissue response to injury, neurons and astrocytes. Based on previous literature, we were expecting a significant response of astrocytes in the subacute phase after stroke, with increased release of EVs in blood as these cells undergo significant activation, known as astrogliosis [38] that causes their molecular, cellular, and functional changes [39,40], starting three days after stroke onset [41]. Indeed, an increase in Glast+ EVs was encountered in comparison with control subjects, but it did not reach statistical significance. Besides, no significant modifications related to neuron-derived vesicles were observed, even though the expression of TSPO on CD171+ was detected.

TSPO is a sensor for brain injury and repair, with very limited expression in the brain under normal physiological conditions, and with dramatic increase after brain injury and inflammation [42]. Although we were expecting to find TSPO on astrocyte-derived and microglia-derived EVs, we found increased expression of TSPO in neuron-derived (CD171+) and microglia (IB4+), but not on EVs from astrocyte (Glast+) nor on EVs from activated microglia (CD11b+). The TSPO selective ligand PK-11195 was also able to bind endothelial-derived EVs (both CD106+ and CD31+), which are known to be the major body cell type expressing this protein, also in the normal brain [43]. The overexpression of TSPO might be due to the severe damage suffered by blood vessels during ischemic stroke, that results in the activation of endothelial cells during the acute and subacute phases of ischemic stroke [44]. The activation state together with the disruption of the blood-brain barrier can actually represent the cause of the increased levels of EVs originating from endothelial cells in the considered population of stroke patients. The other mediator of regenerative stimuli analyzed in the present study is VEGFR2 expression. VEGFR2 plays a fundamental role in cerebral vasculature angiogenesis [45], which has been demonstrated to occur in patients after brain ischemia [46]. Although the soluble form of VEGFR2 was not found to be significantly increased in the serum of subacute stroke patients, compared to CTRL, our data demonstrate an increased expression of VEGFR2 on microglia-derived EVs, providing hints to further investigate the involvement of the VEGFR2 signaling pathway in the cross-communication between microglia cells and to evaluate its ability to be informative about the severity of the suffered ischemic damage and also about the potentiality of recovery.

It has to be noted that EVs are complex carriers of biomolecular moieties and the biomarkers selected in the present paper represent only some of the potential EV components involved in stroke recovery that might also include, for example, EV-associated miRNAs. Further studies are needed to confirm the present observations on a wider cohort of stroke patients and to monitor the expression of the selected biomarkers before and after rehabilitation.

## 4. Materials and Methods

### 4.1. Patients Recruitment and Samples Collection

In this study 19 stroke patients and 20 CTRL were recruited at IRCCS Santa Maria Nascente Operative Recovery and Functional Re-education Unite of Fondazione Don Carlo Gnocchi (FDG; Milan, IT) after protocol approval by Fondazione Don Carlo Gnocchi Ethical Committee (protocol number: Prot.n.20/2018/CE_FdG/FC/SA). Following the declarations of Helsinki, all participants or their representatives provided written informed consent. Patients were enrolled in the subacute phase after the stroke event because this is the timeframe when the rehabilitation program is started. As inclusion criteria, diagnosis of ischemic stroke, no alteration in level of consciousness, ischemia of the intracerebral vessels and supra-aortic trunks or cardioembolism and over 50 years old were required. Patients with a diagnosis of hemorrhagic stroke, state of coma or minimal consciousness, mechanical or systemic arterial thrombolytic therapy, oncological diseases, diseases of the immune and hematological system and neurodegenerative diseases were excluded from the study. Patients were profiled using the MBI [21] clinical scale at hospital admission and at discharge, i.e., after completing the assigned rehabilitation protocol. The rehabilitation protocol included intensive motor and cognitive rehabilitation, with rehabilitation sessions every day for about 2 months (Figure 1A). Control subjects were selected among volunteers in good health status, with no previous diagnosis of ischemia, myocardial infarction, oncologic diseases or other severe systemic or neurologic disorder. Recruited control subjects were age and gender-matched with stroke patients (Table 1). For all recruited subjects, at hospital admission, 10 mL of blood was collected and serum was obtained after 10 min of centrifugation at 2500× *g* at room temperature. Serum samples were aliquoted in cryovials, anonymized and stored at −80 °C until use. Figure 1A shows the schematic representation of the experimental setting of the present study.

### 4.2. Enzyme-Linked Immunosorbent Assay on Serum Samples

Inflammatory cytokines (TNF-α, IL-6, IL-10, ICAM-1/CD54, Fas), Brain derived Neurotrophic factor (BDNF) and the antiaging protein Klotho were quantified in stroke patients (*n* = 19) and CTRL samples (*n* = 16; four control samples could not be analyzed due to limited serum sample volumes). Enzyme-linked immunosorbent assays (ELISA) were performed to detect the presence and the concentration of ICAM-1/CD54 (Human ICAM-1/CD54 Quantikine ELISA Kit—R&D Systems, Biotechne, Minneapolis, MN, USA) and Klotho (Human soluble α-Klotho Assay Kit—Immuno-Biological Laboratories, Minneapolis, MN, USA). Serum samples were diluted 1:2 or 1:20, depending on the kit, and optical intensity was measured within 30 min at 450 nm with Clariostar microplate reader. ELLA (Biotechne) is an instrument that allows automated ELISA assays in a microfluidic Simple Plex cartridge, as a pre-kitted immunoassay based on microfluidic channels and three Glass Nano Reactors for triplicate analyses. ELLA was used to analyze TNF-α, IL-6, IL-10, BDNF, Leptin, Fas and VEGFR2. Following the manufacturer’s instructions, serum samples were diluted 1: 2 or 1:10 directly into the wells of the cartridge and 1 mL of phosphate-buffered saline (PBS) was added for washes. Data were analyzed by Origin2021 (OriginLab, Northampton, MA, USA).

### 4.3. Isolation and Characterization of Extracellular Vesicles

EVs were isolated from the serum of ischemic stroke patients and healthy controls by SEC using qEV Size Exclusion Columns (Izon Science, Christchurch, New Zealand) as previously described [18,24]. Briefly, 500 μL of centrifuged serum were loaded in a column, and the eluted fractions from 7 to 11 were collected and stored at −20 °C in PBS) with protease inhibitor cocktail (Merck, Darmstadt, DA, Germany) until use.

The total protein content of EV samples was measured by BCA assay (Pierce BCA assay kit, Thermo Fisher Scientific, Waltham, MA, USA) after EV lysis (RIPA buffer 1X addition followed by a sequence of vortexing and sonicating for 5 min each, repeated three times). To evaluate the size distribution and the concentration of the isolated EVs, the NTA was carried out thanks to the NanoSight NS300 instrument (Malvern Panalytical, Malvern, UK). Briefly, all EV samples were diluted 1:10 in fresh filtered PBS and then injected for the NTA analysis. Recordings of the movements of particles were collected for 60 s, five times for each sample.

To verify the typical morphology of EVs, TEM images were obtained. In brief, 5 μL of freshly isolated EVs were incubated on Formvar carbon-coated grids. The grid was stained with 2% uranyl acetate solution for 10 min and dried. The sample was then analyzed with TEM (Zeiss, Oberkochen, DE, Germany) at 80 kV.

Reported markers of small EVs (Flotillin-1—BD Transduction Laboratories™, San Jose, CA, USA; CD9—eBioscience, Inc., San Diego, CA, USA; CD81—clone 1.3.3.22, Thermo Fisher Scientific) were evaluated by Western blot. Antibody anti-Albumin (Cell Signaling Technology, Danvers, MA, USA) was also used to evaluate the presence of albumin in the EV preparations. Stroke and CTRL samples were obtained by pooling EV preparations from three stroke patients and three healthy controls, respectively. In brief, freshly isolated EVs were ultra-centrifuged at 4 °C at 100,000× *g* for 70 min (L7-65; Rotor SW60; Beckman Coulter, Brea, CA, USA), lyzed with RIPA buffer and then concentrated using the Pierce SDS-Page Sample-prep Kit following the manufacturer’s protocol (Thermo Fisher Scientific). The secondary HRP conjugated Ab used was goat anti-mouse (Thermo Fisher Scientific) and the protein revelation was performed with LiteAblot TURBO Working Solution (EuroClone, Milan, Italy).

### 4.4. SPRi Analysis

#### 4.4.1. Biosensor Preparation and EV Detection

The SPRi biochip was prepared as previously described [18]. Briefly, the gold surface of the biochip (Horiba, Scientific SAS, Palaiseau, France) was coated with a self-assembled monolayer (SAM) in order to conjugate multiple ligands: Annexin V recombinant protein (VWR, PRSI90-218, MI, Milano, Italy), anti-EphrinB (HBM-EPH-50, HansaBioMed, Tallinn, Estonia), anti-CD171/L1CAM (14-1719-82, eBioscience), IB4 lectin (from Bandeiraea simplicifolia; L3019, Merck KGaA, Darmstadt, Germany), anti-Glast (EAAT1/GLAST-1/SLC1A3 Antibody, NB100-1869SS, Novus Biologicals LLC, Centennial, CO, USA), anti-CD31 (11-0311-81, eBioscience), anti-Klotho (R&D Systems), anti-CD9 (14-0098, eBioscience), anti-CD106 (MA5-16429, Invitrogen, Waltham, MA, USA), anti-CD11b (553311, BD Biosciences, San Jose, CA, USA) for EVs detection, and anti- α-Lactalbumin (GTX77275, GeneTex, Inc., Alton Pkwy, CA, USA) and anti-IgG (407402, BioLegend, Inc., San Diego, CA, USA) to test the functionality of the chip and as a negative control, respectively. Spotting of ligands on the chip surface was performed using the SPRi Arrayer (Horiba Scientific SAS, Palaiseau, France) equipped with a metal-ceramic capillary pin of 0.7 mm diameter for direct contact spotting procedure. Four spots per ligand were obtained for a total of 48 spots (Figure 1C). The biochip was loaded for the SPRi measurements in the XelPleX instrument (Horiba Scientific SAS); 200 µL of sucrose (3 mg/mL) were injected at a flow rate of 50 µL/min for calibration before each experiment; 500 μL of EVs (40 µg/mL measured as total protein content by BCA) in PBS were injected into the SPRi flow chamber with a flow rate of 25 μL/min. SPRi data related to the relative amount of specific EV families were collected on each ligand. The SPRi signals were collected and analyzed by using EzSuite and OriginLab software. SPRi sensorgrams were obtained by subtracting the signal related to the anti-IgG antibody spotted in parallel on the biosensor surface.

#### 4.4.2. Gold Nanoparticle Preparation and Secondary EV Labelling

The presence of the Vascular Endothelial Growth Factor Receptor 2 (VEGFR2) on the surface of the immobilized EVs was evaluated by injecting in SPRi 500 µL of spherical GNPs conjugated with anti-VEGFR2 with a flow rate of 25 µL/min. GNPs of 14 nm were synthesized and functionalized as described by Sguassero et al. [47]. GNPs were used in order to increase the signal intensity as they are surface plasmon-assisted field amplifiers and intrinsic refractive index sensors representing a powerful tool thanks to their rich surface chemistry and high electron density (see Supplementary Material, Figures S1 and S2). Moreover, the presence of a neuroinflammatory marker, Translocator Protein (TSPO) on the EV surface was evaluated by injecting 200 µL of its relative ligand PK11195 (Merck KGaA) with a flow rate of 25 µL/min. At the end of each experiment, the biochip was regenerated with 200 µL of 100 mM NaOH at 50 µL/min. The SPRi signals of each injection were collected and analyzed by using EzSuite software and Origin2021. Sensorgrams were corrected by subtracting the signal related to the negative control (anti-rat IgG antibody) spotted on the chip in order to remove the background noise due to non-specific binding.

### 4.5. Statistical Analysis

Statistical analysis was performed using Origin2021 and SPSS v.28. The Mann–Whitney non-parametric test was used to compare continuous variables obtained from the serum (ELLA and ELISA data) and EV characterization between stroke and CTRL groups. Correlation analysis was performed between SPRi-data and functional independence measured by the MBI score at admission and at discharge from intensive rehabilitation. In particular, either Pearson’s or Spearman’s tests were adopted conditioned to the normality test results (Shapiro–Wilk). The level of significance α was set at *p*-value = 0.05.

Then, features significantly correlating with either the admission or discharge MBI, entered a five-fold cross-validated multivariate regularized linear regression (Elastic-Net, EN) with outcome, respectively, set to the admission or discharge MBI. Regularization is adopted to prevent the risk of overfitting. EN is a regularized method that linearly combines the regularization penalties of the LASSO and Ridge regression limiting the respective limitations of the two methods as used in isolation [48]. Ridge adds to linear regression models quadratic regularization ($\lambda_{2}{\left|\left|\beta \right|\right|}^{2}$): it always assigns a non-zero coefficient to all features in the model, consequently failing in eliminating coefficients even if the corresponding independent variable is irrelevant to the prediction. Conversely, LASSO regularization term ($\lambda_{1}{\left|\left|\beta \right|\right|}_{1})$ fosters the neglect of specific features but is known to suffer when the dimensionality of the dataset is higher than the number of the available examples or when multicollinear independent variables are present [49,50]. The weighted use of both regularization terms results in regression parameters β estimates as follows:

$$\beta =argmin_{\left\{\beta \right\}}{\left|\left|y-X\beta \right|\right|}^{2}+\lambda_{2}{\left|\left|\beta \right|\right|}^{2}+\lambda_{1}{\left|\left|\beta \right|\right|}_{1}$$

where *X* is the feature set and *y* is the dependent variable. Regularization parameters tune the type of regularization applied, with the special cases $\lambda_{2}$ = 0, $\lambda_{1}\neq$ 0 and $\lambda_{2}\neq$ 0, $\lambda_{1}=$ 0 corresponding to the LASSO and Ridge regression, respectively. The median absolute value and interquartile range (IQR) between actual and predicted values will be used to evaluate the multivariate accuracy of the two models together with the R^2^ coefficient. Regression analyses and cross-validation were conducted using Python libraries (statamodels, sklearn).

## 5. Conclusions

Taken together, our results suggest that microglia-derived EVs and endothelial EVs are significantly involved in the intercellular communications that occur more than 10 days after ischemic stroke. The reported data suggest that EVs could be even more effective than previously proposed biomarkers in the profiling of patients in the subacute phase after ischemic stroke and in the prediction of their recovery after rehabilitation. Although our results are valid for mild ischemic stroke patients over 50 years old, our data support the idea that EVs are involved in crucial mechanisms in post-stroke brain remodeling that could be further explored and enhanced to favor patients’ recovery. Despite the expected variability of data from patients after ischemic stroke due to lesion extent and spontaneous repair processes, EVs seem to retain sufficient information for the biochemical profiling of patients. This work provides hints and methodology (multiplexing SPRi based biosensor) to further deepen the role of EVs as biomarkers of rehabilitation after ischemic stroke and as key players in stroke recovery, to be used as decision support systems for clinicians and physiotherapists dealing with complex patients’ profiles in the subacute phase of the disease.

## Figures and Tables

**Table 1:** General features of the ischemic stroke patients (Stroke) and control subjects (Control) enrolled in the present study. In parenthesis, median and interquartile ranges are reported for numerical variables whilst count and percentages are reported for categorical variables.

Independent Variables	Stroke (*n* = 19)	Control (*n* = 20)
**Age**, years	74 (18)	66 (8)
**Sex**, female	8 (42.1%)	10 (50.0%)
**Modified Barthel Index admission**, points	30 (26)	--
**Modified Barthel Index at discharge**, points	56 (54)	--
**Time post onset**, days	21 (14)	--
**Relapsing patients**, number	8 (42.1%)	--
**Patients with diabetes**, number	10 (62.6%)	--

**Table 2:** Univariate correlation analysis between SPRi instrumental variables and MBI at admission and discharge. Only variables that showed significant correlation with MBI at admission and/or discharge are reported. Significant correlations are reported in bold.

	Independent Variables	MBI at Admission		MBI at Discharge	
		R^2^	*p*	R^2^	*p*
SPRi	CD11b+	0.694	0.056	**0.881**	**0.004**
	VEGFR2 on CD11b+	**−0.680**	**0.044**	−0.644	0.061
	VEGFR2 on IB4+	−0.244	0.330	**−0.563**	**0.015**
	TSPO on CD106+	**0.671**	**0.048**	0.402	0.283
	TSPO on CD31+	**0.487**	**0.035**	**0.694**	**0.001**
	TSPO on CD9+	**0.458**	**0.049**	0.258	0.287

## Data Availability

All data generated or analyzed during this study are included in this published article and in the Supplementary Materials.

## Supplementary Materials

The following supporting information can be downloaded at: https://www.mdpi.com/article/10.3390/ijms24097937/s1. References [47,51,52] are cited in the Supplementary Materials.

## References

[1] Bull F., Goenka S., Lambert V., Pratt M. (2017). Physical Activity for the Prevention of Cardiometabolic Disease. Disease Control Priorities, Third Edition (Volume 5): Cardiovascular, Respiratory, and Related Disorders.

[2] Taricco M., Cecchi F., Cassio A., Lavezzi S., Scarponi F., Montis A., Gatta G., Bernucci C., Franceschini M., Paolucci S. (2020). PMIC 2020 Protocollo Di Valutazione Riabilitativa Di Minima Della Persona Con Ictus Cerebrale Versione 2020. Soc. Ital. Med. Fis..

[3] Gualerzi A., Picciolini S., Rodà F., Bedoni M. (2021). Extracellular Vesicles in Regeneration and Rehabilitation Recovery after Stroke. Biology.

[4] Bembenek J.P., Kurczych K., Kłysz B., Cudna A., Antczak J., Członkowska A. (2020). Prediction of Recovery and Outcome Using Motor Evoked Potentials and Brain Derived Neurotrophic Factor in Subacute Stroke. J. Stroke Cerebrovasc. Dis..

[5] Gandolfi M., Smania N., Vella A., Picelli A., Chirumbolo S. (2017). Assessed and Emerging Biomarkers in Stroke and Training-Mediated Stroke Recovery: State of the Art. Neural Plast..

[6] Carandina A., Favero C., Sacco R.M., Hoxha M., Torgano G., Montano N., Bollati V., Tobaldini E. (2022). The Role of Extracellular Vesicles in Ischemic Stroke Severity. Biology.

[7] Dewdney B., Trollope A., Moxon J., Thomas Manapurathe D., Biros E., Golledge J. (2018). Circulating MicroRNAs as Biomarkers for Acute Ischemic Stroke: A Systematic Review. J. Stroke Cerebrovasc. Dis..

[8] Deng Y., Huang P., Zhang F., Chen T. (2022). Association of MicroRNAs With Risk of Stroke: A Meta-Analysis. Front. Neurol..

[9] Fullerton J.L., Cosgrove C.C., Rooney R.A., Work L.M. (2022). Extracellular Vesicles and Their MicroRNA Cargo in Ischaemic Stroke. J. Physiol..

[10] Erdbrügger U., Lannigan J. (2016). Analytical Challenges of Extracellular Vesicle Detection: A Comparison of Different Techniques: Analytical Challenges of Extracellular Vesicle Detection. Cytometry.

[11] Gualerzi A., Picciolini S., Carlomagno C., Rodà F., Bedoni M. (2021). Biophotonics for Diagnostic Detection of Extracellular Vesicles. Adv. Drug Deliv. Rev..

[12] Hartjes T., Mytnyk S., Jenster G., van Steijn V., van Royen M. (2019). Extracellular Vesicle Quantification and Characterization: Common Methods and Emerging Approaches. Bioengineering.

[13] Palmieri V., Lucchetti D., Gatto I., Maiorana A., Marcantoni M., Maulucci G., Papi M., Pola R., De Spirito M., Sgambato A. (2014). Dynamic Light Scattering for the Characterization and Counting of Extracellular Vesicles: A Powerful Noninvasive Tool. J. Nanopart. Res..

[14] Royo F., Théry C., Falcón-Pérez J.M., Nieuwland R., Witwer K.W. (2020). Methods for Separation and Characterization of Extracellular Vesicles: Results of a Worldwide Survey Performed by the ISEV Rigor and Standardization Subcommittee. Cells.

[15] Serrano-Pertierra E., Oliveira-Rodríguez M., Matos M., Gutiérrez G., Moyano A., Salvador M., Rivas M., Blanco-López M.C. (2020). Extracellular Vesicles: Current Analytical Techniques for Detection and Quantification. Biomolecules.

[16] Sharma S., LeClaire M., Gimzewski J.K. (2018). Ascent of Atomic Force Microscopy as a Nanoanalytical Tool for Exosomes and Other Extracellular Vesicles. Nanotechnology.

[17] Wang J., Kao Y., Zhou Q., Wuethrich A., Stark M.S., Schaider H., Soyer H.P., Lin L.L., Trau M. (2022). An Integrated Microfluidic-SERS Platform Enables Sensitive Phenotyping of Serum Extracellular Vesicles in Early Stage Melanomas. Adv. Funct. Mater..

[18] Picciolini S., Gualerzi A., Vanna R., Sguassero A., Gramatica F., Bedoni M., Masserini M., Morasso C. (2018). Detection and Characterization of Different Brain-Derived Subpopulations of Plasma Exosomes by Surface Plasmon Resonance Imaging. Anal. Chem..

[19] Théry C., Witwer K.W., Aikawa E., Alcaraz M.J., Anderson J.D., Andriantsitohaina R., Antoniou A., Arab T., Archer F., Atkin-Smith G.K. (2018). Minimal Information for Studies of Extracellular Vesicles 2018 (MISEV2018): A Position Statement of the International Society for Extracellular Vesicles and Update of the MISEV2014 Guidelines. J. Extracell. Vesicles.

[20] Lasek-Bal A., Jędrzejowska-Szypułka H., Różycka J., Bal W., Holecki M., Duława J., Lewin-Kowalik J. (2015). Low Concentration of BDNF in the Acute Phase of Ischemic Stroke as a Factor in Poor Prognosis in Terms of Functional Status of Patients. Med. Sci. Monit..

[21] Shah S., Vanclay F., Cooper B. (1989). Improving the Sensitivity of the Barthel Index for Stroke Rehabilitation. J. Clin. Epidemiol..

[22] Sahu A., Mamiya H., Shinde S.N., Cheikhi A., Winter L.L., Vo N.V., Stolz D., Roginskaya V., Tang W.Y., Croix C.S. (2018). Age-Related Declines in α-Klotho Drive Progenitor Cell Mitochondrial Dysfunction and Impaired Muscle Regeneration. Nat. Commun..

[23] Lee J.B., Woo H.G., Chang Y., Jin Y.M., Jo I., Kim J., Song T.J. (2019). Plasma Klotho Concentrations Predict Functional Outcome at Three Months after Acute Ischemic Stroke Patients. Ann. Med..

[24] Picciolini S., Gualerzi A., Carlomagno C., Cabinio M., Sorrentino S., Baglio F., Bedoni M. (2021). An SPRi-Based Biosensor Pilot Study: Analysis of Multiple Circulating Extracellular Vesicles and Hippocampal Volume in Alzheimer’s Disease. J. Pharm. Biomed. Anal..

[25] Tallon C., Picciolini S., Yoo S., Thomas A.G., Pal A., Alt J., Carlomagno C., Gualerzi A., Rais R., Haughey N.J. (2021). Inhibition of Neutral Sphingomyelinase 2 Reduces Extracellular Vesicle Release from Neurons, Oligodendrocytes, and Activated Microglial Cells Following Acute Brain Injury. Biochem. Pharmacol..

[26] Dimitrova-Shumkovska J., Krstanoski L., Veenman L. (2020). Diagnostic and Therapeutic Potential of TSPO Studies Regarding Neurodegenerative Diseases, Psychiatric Disorders, Alcohol Use Disorders, Traumatic Brain Injury, and Stroke: An Update. Cells.

[27] Zhang Z.G., Buller B., Chopp M. (2019). Exosomes—Beyond Stem Cells for Restorative Therapy in Stroke and Neurological Injury. Nat. Rev. Neurol..

[28] Yousif G., Qadri S., Haik M., Haik Y., Parray A.S., Shuaib A. (2021). Circulating Exosomes of Neuronal Origin as Potential Early Biomarkers for Development of Stroke. Mol. Diagn. Ther..

[29] Bitencourt A.C.S., Timóteo R.P., Bazan R., Silva M.V., Filho L.G.d.S., Ratkevicius C.M.A., de Assunção T.S.F., de Oliveira A.P.S., Luvizutto G.J. (2022). Association of Proinflammatory Cytokine Levels with Stroke Severity, Infarct Size, and Muscle Strength in the Acute Phase of Stroke. J. Stroke Cerebrovasc. Dis..

[30] Wytrykowska A., Prosba-Mackiewicz M., Nyka W.M. (2016). IL-1β, TNF-α, and IL-6 Levels in Gingival Fluid and Serum of Patients with Ischemic Stroke. J. Oral Sci..

[31] Intiso D., Zarrelli M.M., Lagioia G., Rienzo F.D., Ambrosio C.C.D., Simone P., Tonali P., Cioffi R.P. (2004). Tumor Necrosis Factor Alpha Serum Levels and Inflammatory Response in Acute Ischemic Stroke Patients. Neurol. Sci..

[32] Wu B.N., Wu J., Hao D.L., Mao L.L., Zhang J., Huang T.T. (2018). High Serum SICAM-1 Is Correlated with Cerebral Microbleeds and Hemorrhagic Transformation in Ischemic Stroke Patients. Br. J. Neurosurg..

[33] Delgado P., Cuadrado E., Rosell A., Alvarez-Sabin J., Ortega-Aznar A., Hernandez-Guillamon M., Penalba A., Molina C.A., Montaner J. (2008). Fas System Activation in Perihematomal Areas after Spontaneous Intracerebral Hemorrhage. Stroke.

[34] Couch Y., Akbar N., Davis S., Fischer R., Dickens A.M., Neuhaus A.A., Burgess A.I., Rothwell P.M., Buchan A.M. (2017). Inflammatory Stroke Extracellular Vesicles Induce Macrophage Activation. Stroke.

[35] Santos G.L., Alcântara C.C., Silva-Couto M.A., García-Salazar L.F., Russo T.L. (2016). Decreased Brain-Derived Neurotrophic Factor Serum Concentrations in Chronic Post-Stroke Subjects. J. Stroke Cerebrovasc. Dis..

[36] Zhou H.J., Li H., Shi M.Q., Mao X.N., Liu D.L., Chang Y.R., Gan Y.M., Kuang X., Du J.R. (2018). Protective Effect of Klotho against Ischemic Brain Injury Is Associated with Inhibition of RIG-I/NF-ΚB Signaling. Front. Pharmacol..

[37] Sahu A., Clemens Z.J., Shinde S.N., Sivakumar S., Pius A., Bhatia A., Picciolini S., Carlomagno C., Gualerzi A., Bedoni M. (2021). Regulation of Aged Skeletal Muscle Regeneration by Circulating Extracellular Vesicles. Nat. Aging.

[38] Bonaventura A., Liberale L., Vecchié A., Casula M., Carbone F., Dallegri F., Montecucco F. (2016). Update on Inflammatory Biomarkers and Treatments in Ischemic Stroke. Int. J. Mol. Sci..

[39] Roy Choudhury G., Ryou M.-G., Poteet E., Wen Y., He R., Sun F., Yuan F., Jin K., Yang S.-H. (2014). Involvement of P38 MAPK in Reactive Astrogliosis Induced by Ischemic Stroke. Brain Res..

[40] Brenna S., Altmeppen H.C., Mohammadi B., Rissiek B., Schlink F., Ludewig P., Krisp C., Schlüter H., Failla A.V., Schneider C. (2020). Characterization of Brain-derived Extracellular Vesicles Reveals Changes in Cellular Origin after Stroke and Enrichment of the Prion Protein with a Potential Role in Cellular Uptake. J. Extracell. Vesicles.

[41] Barthels D., Das H. (2020). Current Advances in Ischemic Stroke Research and Therapies. Biochim. Biophys. Acta Mol. Basis Dis..

[42] Chen M.-K., Guilarte T.R. (2008). Translocator Protein 18 KDa (TSPO): Molecular Sensor of Brain Injury and Repair. Pharmacol. Ther..

[43] Betlazar C., Harrison-Brown M., Middleton R.J., Banati R., Liu G.-J. (2018). Cellular Sources and Regional Variations in the Expression of the Neuroinflammatory Marker Translocator Protein (TSPO) in the Normal Brain. Int. J. Mol. Sci..

[44] Kozuka K., Kohriyama T., Ikeda J., Nakamura S., Nomura E., Kajikawa H. (2002). Endothelial Markers and Adhesion Molecules in Acute Ischemic Stroke-Sequential Change and Differences in Stroke Subtype. Atherosclerosis.

[45] Pianta S., Lee J.Y., Tuazon J.P., Castelli V., Mantohac L.M., Tajiri N., Borlongan C.V. (2019). A Short Bout of Exercise Prior to Stroke Improves Functional Outcomes by Enhancing Angiogenesis. Neuromol. Med..

[46] Liu J. (2015). Poststroke Angiogenesis: Blood, Bloom, or Brood?. Stroke A J. Cereb. Circ..

[47] Sguassero A., Artiga Á., Morasso C., Jimenez R.R., Rapún R.M., Mancuso R., Agostini S., Hernis A., Abols A., Linē A. (2019). A Simple and Universal Enzyme-Free Approach for the Detection of Multiple MicroRNAs Using a Single Nanostructured Enhancer of Surface Plasmon Resonance Imaging. Anal. Bioanal. Chem..

[48] Zou H., Hastie T. (2005). Regularization and Variable Selection via the Elastic Net. J. R. Statistical. Soc. B.

[49] Fan J., Li R. Statistical Challenges with High Dimensionality: Feature Selection in Knowledge Discovery. Proceedings of the International Congress of Mathematicians.

[50] Yamada M., Koh T., Iwata T., Shawe-Taylor J., Kaski S. Localized Lasso for High-Dimensional Regression. Proceedings of the 20th International Conference on Artificial Intelligence and Statistics.

[51] Frens G. (1973). Controlled Nucleation for the Regulation of the Particle Size in Monodisperse Gold Suspensions. Nat. Phys. Sci..

[52] Ojea-Jiménez I., Bastús N.G., Puntes V. (2011). Influence of the Sequence of the Reagents Addition in the Citrate-Mediated Synthesis of Gold Nanoparticles. J. Phys. Chem. C.

