# Supplementary material for: Multiplexing Biosensor for the Detection of Extracellular Vesicles as Biomarkers of Tissue Damage and Recovery after Ischemic Stroke
Source: Int J Mol Sci. 2023 Apr 27;24(9):7937. doi: 10.3390/ijms24097937 (PMC10177901; doi:10.3390/ijms24097937)
Supplement: Supplementary file 1 [file ijms-24-07937-s001.zip › ijms-2325072-supplementary.pdf]

Supplementary Materials

Multiplexing Biosensor for the Detection of Extracellular Vesicles as Biomarkers of Tissue Damage and Recovery after Ischemic Stroke

Silvia Picciolini[1], Valentina Mangolini[1,2], Francesca Rodà[1,3], Angelo Montesano[1], Francesca Arnaboldi[4], Piergiuseppe Liuzzi[5,6], Andrea Mannini[5], Marzia Bedoni[1] and Alice Gualerzi[1,*]

1 IRCCS Fondazione Don Carlo Gnocchi ONLUS, 20148 Milano, Italy
2 Dipartimento di Medicina Molecolare e Traslazionale, Università degli Studi di Brescia, 25122 Brescia, Italy
3 Clinical and Experimental Medicine PhD Program, University of Modena and Reggio Emilia, 42100 Modena, Italy
4 Dipartimento di Scienze Biomediche per la Salute, Università degli Studi di Milano, 20133 Milano, Italy
5 IRCCS Fondazione Don Carlo Gnocchi ONLUS, 50143 Firenze, Italy
6 Scuola Superiore Sant'Anna, Istituto di BioRobotica, 56025 Pontedera, Italy

* Correspondence: agualerzi@dongnocchi.it; Tel.: +39-0240308533

1. Gold nanoparticles synthesis and functionalization

The synthesis of 14 nm Gold Nanoparticles (GNPs) with maximum OD at 520 nm was performed following the Frens method [51,52]. 300 ml of microfiltered H2O were brought to a boil, 12 ml of sodium citrate (50 mM) were added to H_2O and stirred for 15 minutes. 2.5 ml of tetrachlorauric acid (20 mM) were added to the solution at boiling condition and stirred for about an hour. The solution was cooled down through stirring for 2 hours at room temperature.

GNPs were firstly functionalized with neutravidin according to the protocol described by Sguassero et al.[47]. Briefly, GNPs (10 nM) were mixed with SDS (0.028% w/v) and PEG (SH-C11-EG6-OCH$_2$- COOH, ProChimia Surfaces (Sopot, PL), 20 μM) that allows nanoparticles functionalization thanks to the carboxylic (-COOH) and the thiolic group (-SH). Then, NaOH was added to have a final concentration of 25 mM and the solution was gently stirred for 16 hours at room temperature. In order to eliminate the excess of PEG, washing steps with water were performed. The PEG carboxylic groups of the GNPs were activated using EDC/sulfo-NHS solution for 15 minutes, centrifuged at 14,000 g for 20 minutes and resuspended in 880 μl of phosphate buffer (1 mM, pH 5.2). Different amounts of neutravidin (280 μg/ml; 0, 2, 4, 6, 10, 15, 20 μl) were added to the mixture that was gently stirred for 20 minutes and 160 μl of taurine (100 mM in sodium borate buffer) were added. Lastly, neutravidin coated GNPs (nGNPs) were resuspended resuspended in 20 μl of sucrose (20 mg/ml) and agarose gel (0.6% in TBE 0.5M) electrophoresis was performed for 30 minutes at 225V in TBE (0.5, pH 8) in order to verify the binding of neutravidin. The results confirmed that higher concentrations of neutravidin correlate with lower migration rates showing that neutravidin volumes lower than 6 μl do not allow a total functionalization of the GNPs while neutravidin volumes higher than 10 μl do (Figure S1). Indeed, the volume of 10 μl was selected for GNP functionalization as it was the lowest neutravidin volume to guarantee an optimal conjugation of all GNPs.

Then, nGNPs were diluted in PBS (10 mM, pH 7.4) to obtain a final absorbance of 0.25 OD at 524 nm measured with Nanodrop instrument. Lastly, the biotinylated ligands (antibody anti-Klotho (Biotechne, BAF1819 and antibody anti-VEGFR-2 (Biotechne, NB200-208B) were mixed with nGNPs to have the final concentration of 5 nM.

2. Enhanced SPRi optimization

The optimization of the enhanced SPRi on different EV families was performed using nGNPs functionalized with Ab anti-CD9, a general marker of EVs. EVs analysed in this experiment were isolated from the serum of

a healthy control subject. We found that the nGNPs functionalized with Ab anti-CD9 gave a significant SPRi signal on all EV families, interacting with the membrane antigen of previously immobilized EV families (Figure S2). The specificity of this signal was demonstrated by testing three different control cases: nGNPs, Ab anti-CD9 and biotinylated Ab anti-CD9, injected as second analytes. Indeed, the nGNPs caused SPRi signals with very low intensities that are considered negligible in Glast+, Klotho+, Annexin-V+, IB4+, Ephrin B+ EV families, and only a more intense signal on CD31+ EV family compared to the others. Furthermore, it did not induce an SPRi signal on CD171+ EV population. The biotinylated and non-biotinylated Ab anti-CD9 did not cause a relevant shift of the SPR angle in any of the EV populations. Those results showed that nGNPs functionalized with Ab anti-CD9 allow the identification of CD9 antigens on EV membrane through the high and specific signals on all EV samples, which is what we expected, as CD9 is a known general marker of EVs.

Indeed, signals obtained with the control cases were reduced and negligible compared to the ones related to Ab-conjugated GNPs, thus allowing the initiation of the study by using this optimized strategy for the enhancement of SPRi signal intensities to detect specific EV membrane antigens.

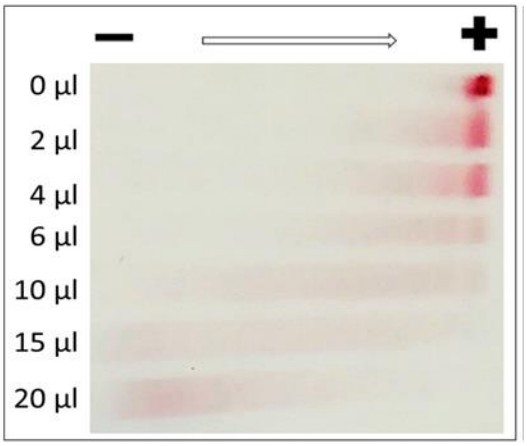

Figure S1. Electrophoretic run of GNPs functionalized with increasing volume of neutravidin 280 µg/ml.

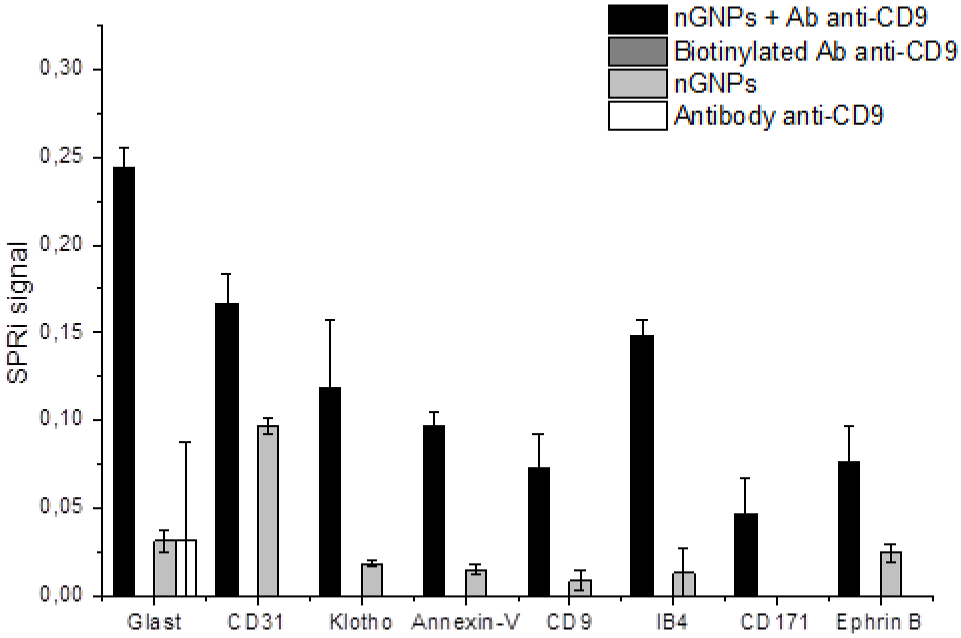

Figure S2. SPRi signal intensities (mean and standard deviation) of nGNPs functionalized with Ab anti-CD9 and three control cases (nGNPs, Ab anti-CD9 and biotinylated Ab anti-CD9) normalized for the intensities of signals related to EV injection.

Table S1. Experimental mean value ± standard deviation obtained for the quantification of soluble mediators of inflammation and regeneration by enzyme-linked immunosorbent assays on serum samples of control samples (CTRL) and stroke patients (STROKE), as shown in Figure 2. For every measurement, the number of analysed subject is reported because the limited serum sample volumes or technical errors did not allow to obtain a result for all samples.

	Stroke		Control	
	Mean ± Std. Deviation	n	Mean ± Std. Deviation	n
**ICAM**, ng/ml	306.78 ± 136.99	17	159 ± 51.55	16
**IL-6**, pg/ml	12.73 ± 9	19	3.13 ± 4.36	16
**IL-10**, pg/ml	4.42 ± 1.71	19	1.48 ± 0.54	16
**TNFa**, pg/ml	20.92 ± 6.71	19	14.03 ± 11.75	16
**Leptin**, ng/ml	37.34 ± 37.67	19	11.64 ± 7.73	15
**Fas**, ng/ml	15.18 ± 5.87	19	12.271 ± 3.67	15
**BDNF**, ng/ml	24.93 ± 9.95	19	32.16 ± 9.31	15
**VEGFR2**, ng/ml	12.06 ± 2.79	19	10.61 ± 2.65	15
**Klotho**, pg/ml	438.92 ± 208.98	19	463.97 ± 226.13	16

Table S2. Experimental mean value ± standard deviation obtained for the quantification of particles by NTA and BCA assays, as shown in Figure 3.

	Stroke	Control
	Mean ± Std. Deviation	Mean ± Std. Deviation
Particles/ml	$4.38x10^9 \pm 2.55x10^9$	$4.15x10^9 \pm 1.92\ x10^9$
μg/particles	$4\ x10^8 \pm 2.19\ x10^8$	$3.24\ x10^8 \pm 1.7\ x10^8$

Table S3. Experimental SPRi intensities (average ± standard error) related to the injection of EVs of 19 ischemic stroke patients and 20 CTRL subjects, as shown in Figure 4.

	Stroke	Control
	Mean ± Std. Error	Mean ± Std. Error
CD106+	2.26 ± 0.46	1.23 ± 0.48
CD31+	1.47 ± 0.31	1.43 ± 0.35
IB4+	1.16 ± 0.17	1.2 ± 0.288
CD11b+	2.33 ± 0.61	1.91 ± 0.26
CD171+	1.42 ± 0.19	1.35 ± 0.19
EphrinB+	1.27 ± 0.24	1.04 ± 0.28
Glast+	1.38 ± 0.27	1.28 ± 0.38
Klotho+	1.6 ± 0.25	1.79 ± 0.36
Annexin V	1.8 ± 0.35	1.81± 0.37

Table S4. Experimental SPRi intensities (average ± standard error) obtained on the EVs immobilized on the biosensor by secondary labelling with VEGFR2 antibody conjugated with GNPs or with the TSPO ligand PK-11195, as shown in Figure 5.

	**Stroke**	**Control**
**VEGFR2 on**	Mean ± Std. Error	Mean ± Std. Error
CD106+	1.33 ± 0.23	3.12 ± 1
CD31+	0.37 ± 0.13	0.78 ± 0.42
IB4+	0.27 ± 0.13	0.26 ± 0.14
CD11b+	0.23 ± 0.12	0.17 ± 0.13
CD171+	0.12 ± 0.07	0.25 ± 0.12
EphrinB+	0.34 ± 0.13	0.43 ± 0.25
Glast+	0.31 ± 0.07	0.86 ± 0.47

	**Stroke**	**Control**
**TSPO on**	Mean ± Std. Error	Mean ± Std. Error
CD106+	0.25 ± 0.18	0.42 ± 0.25
CD31+	0.18 ± 0.11	0.33 ± 0.15
IB4+	0.68 ± 0.2	0.97 ± 0.45
CD11b+	0.65 ± 0.36	0.24 ± 0.05
CD171+	0.79 ± 0.47	0.3 ± 0.08
EphrinB+	0.28 ± 0.12	0.24 ± 0.11
Glast+	0.26 ± 0.1	0.3 ± 0.11
